# Inhibition of Autophagy Negates Radiofrequency-Induced Adaptive Response in SH-SY5Y Neuroblastoma Cells

**DOI:** 10.3390/ijms23158414

**Published:** 2022-07-29

**Authors:** Anna Sannino, Maria Rosaria Scarfì, Mélody Dufossée, Stefania Romeo, Loredana Poeta, Valerie Prouzet-Mauléon, Muriel Priault, Olga Zeni

**Affiliations:** 1 Institute for Electromagnetic Sensing of the Environment (IREA), National Research Council, Via Diocleziano 328, 80124 Napoli, Italy; sannino.a@irea.cnr.it (A.S.); romeo.s@irea.cnr.it (S.R.); poeta.l@irea.cnr.it (L.P.); zeni.o@irea.cnr.it (O.Z.); 2 Univ. Bordeaux, CNRS, IBGC, UMR 5095, F-33000 Bordeaux, France; melody.dufossee@ibgc.cnrs.fr (M.D.); muriel.priault@ibgc.cnrs.fr (M.P.); 3 Plateformecrisp’edit—TBMCore, Université de Bordeaux, F-33000 Bordeaux, France; valerie.prouzet-mauleon@u-bordeaux.fr; 4 INSERM, US005, F-33000 Bordeaux, France; 5 CNRS, UAR3427, F-33000 Bordeaux, France

**Keywords:** RF-electromagnetic fields, in vitro exposure, DNA damage, adaptive response, autophagy, autophagy inhibitors, CRISPR cells

## Abstract

In the last years, radiofrequency (RF) has demonstrated that it can reduce DNA damage induced by a subsequent treatment with chemical or physical agents in different cell types, resembling the adaptive response, a phenomenon well documented in radiobiology. Such an effect has also been reported by other authors both in vitro and in vivo, and plausible hypotheses have been formulated, spanning from the perturbation of the cell redox status, to DNA repair mechanisms, and stress response machinery, as possible cellular mechanisms activated by RF pre-exposure. These mechanisms may underpin the observed phenomenon, and require deeper investigations. The present study aimed to determine whether autophagy contributes to RF-induced adaptive response. To this purpose, SH-SY5Y human neuroblastoma cells were exposed for 20 h to 1950 MHz, UMTS signal, and then treated with menadione. The results obtained indicated a reduction in menadione-induced DNA damage, assessed by applying the comet assay. Such a reduction was negated when autophagy was inhibited by bafilomycin A1 and E64d. Moreover, CRISPR SH-SY5Y cell lines defective for *ATG7* or *ATG5* genes did not show an adaptive response. These findings suggest the involvement of autophagy in the RF-induced adaptive response in human neuroblastoma cells; although, further investigation is required to extend such observation at the molecular level.

## 1. Introduction

Over the last decades, technologies employing radiofrequency (RF) electromagnetic fields (EMF) have emerged, particularly in the telecommunication frequency range. The deployment of 5G networks will increase this tendency and pervade different areas of our daily life. Thus, RF exposure has become unavoidable and can be considered a hallmark of modern society, which provides grounding for increasing public concern as a possible health hazard. Several biological effects have been described after exposure to RF-EMF (100 kHz–300 GHz), and the relevant interactions are discussed in terms of thermal versus non-thermal mechanisms. Tissue heating is the only established effect of exposure to RF-EMF, for which exposure limits have been defined [1]. On the contrary, non-thermal mechanisms have not yet been clarified. Many hypotheses have been suggested, but none of them have been proven so far to substantiate the biological and health effects across different research domains, from experimental studies to epidemiological research [2,3,4,5].

More specifically, by considering in vitro investigations, which are the majority of the studies available in the literature on this topic, several biological effects have been reported on different cell models by measuring many cellular endpoints [6,7,8]. In addition, several studies addressed the effects of RF in combination with other physical and chemical agents. Such effects strictly depend on the cell type, on the RF exposure conditions (frequency, signal, specific absorption rate, exposure duration, and modulation), and on the dose and timing of the agents used, and they can trigger both adverse and beneficial outcomes [9,10]. As far as beneficial effects are concerned, our research group evidenced a phenomenon for which mammalian cells pre-exposed to RF were protected from the damage induced by a subsequent treatment with chemical or physical genotoxic agents, in a process resembling the ionizing radiation-induced Adaptive Response (AR) [11,12,13,14,15,16,17,18,19,20]. Such an effect has also been reported by other authors both in vitro [21,22,23] and in vivo [24,25]. Plausible hypotheses have been formulated as possible mechanisms activated by RF pre-exposure that underpin the observed phenomenon, spanning from the perturbation of the cell redox status [11,26,27] to DNA repair, and stress response machinery [14,21,22,23,25]. Recent studies have reported that RF exposure can activate the autophagic process, providing the rationale for its implication at the onset of RF-induced beneficial effects [28,29,30,31,32]. Moreover, autophagy was recently proposed to be involved in the low-dose ionizing radiation-induced AR [33,34]. 

Autophagy is a highly conserved cytoprotective program throughout eukaryotes, whose purpose is to help cells to rapidly adapt to any challenging situation. It is the first line of defense against stress, whether it is driven by external cues (physical, nutritive, or chemical stress or exposure to biological infections), or by intracellular alterations (e.g., protein aggregation). Hence, in response to a wide array of stimuli, the autophagic program, which normally runs at a basal level to warrant intra-cellular quality control, will be up-regulated. Autophagy-related genes (*ATG*) will orchestrate the increased turnover of dispensable cellular components to synthesize those essential for cell survival. Vesicular trafficking will thus be intensified, where portions of cytoplasmic constituents and organelles are enclosed in autophagosomes and routed to release their cargo into lysosomes. The resulting degradation products are further made available to be reused for the synthesis of essential components [35].

The purpose of the present study was to investigate whether autophagy has a role in RF-induced AR. To this aim, SH-SY5Y human neuroblastoma cells were pre-exposed for 20 h to 1950 MHz, UMTS signal, 0.3 W/kg Specific Absorption Rate (SAR) level, and then treated with menadione (MD). These exposure conditions and treatment procedures were exactly the same as in our previous work, which induced AR in the SH-SY5Y cell model [11]. By applying the comet assay, the reduction in the menadione-induced DNA damage was evaluated in SH-SY5Y wild-type cells, in presence and in absence of autophagy inhibitors, as well as in defective *ATG* genes cell lines.

## 2. Results

### 2.1. RF-Induced AR Is Negated in Presence of Inhibitors of Autophagy

We have previously shown that sham-exposed SH-SY5Y cultures in absence and in presence of MD exhibited cell viability and % DNA in the tail comparable with control cultures kept in a standard incubator [11,19]. Therefore, since sham-exposed cells experienced the very same environmental conditions as the exposed ones (except for RF exposure), the former were considered as the most appropriate reference control in all the experiments. Moreover, since the determination of cell survival is critical with respect to the biological significance of comet results [36], cell viability was assayed in all the experimental cultures, and it resulted in higher than 85% (trypan blue dye exclusion method; data not shown).

**Figure 1:**
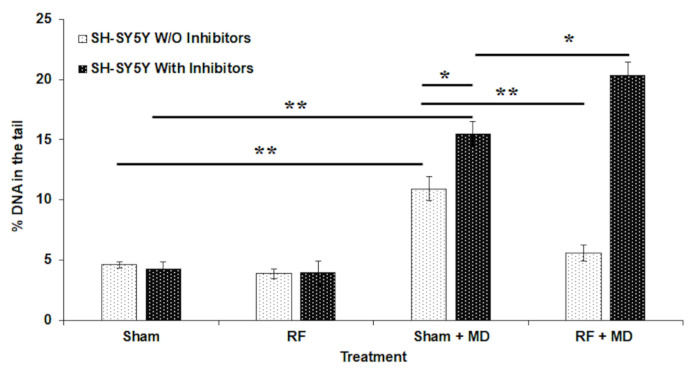
% DNA in the tail in SH-SY5Y cells exposed to RF, 0.3 W/kg SAR, for 20 h, in presence (With) and absence (W/O) of autophagy inhibitors. For each condition, the following samples were analyzed: sham-exposed (Sham), RF-exposed (RF), sham-exposed and treated with MD (Sham + MD), and RF-exposed and treated with MD (RF + MD). Each data point represents the mean ± SE of the 4 independent experiments. * *p* < 0.05; ** *p* < 0.01 (two-tailed unpaired Student’s *t*-test).

Here, we aimed to elucidate whether the inhibition of autophagy interferes with the onset of AR. In Figure 1, we show the % of DNA in the tail in the different experimental cultures in presence and in absence of bafilomycin A1, and E64d inhibitors, as a result of four independent experiments (mean ± SE). 

In absence of inhibitors, we found that RF exposure did not induce DNA damage (RF vs. Sham), whereas, as expected, MD treatment induced a significant increase in DNA damage (36% increase; Sham + MD vs. Sham, *p* < 0.01). Pre-exposure to RF was able to induce a statistically significant reduction in the MD-induced damage (49%; RF + MD vs. Sham + MD, *p* < 0.01), confirming the induction of AR in this cell model [11,19].

The presence of autophagy inhibitors did not alter the levels of DNA damage in Sham/RF-exposed cultures (Sham + inhibitors vs. Sham W/O inhibitors; RF + inhibitors vs. RF W/O inhibitors; RF vs. Sham; *p* > 0.05 in all cases). This result allows us to confirm that the autophagy inhibitors are not toxic at the concentrations we used.

In cultures treated with autophagy inhibitors, AR was negated, and instead, an additive effect was recorded in RF-exposed and MD-treated cultures compared to sham-exposed and MD-treated ones (30% increase in RF + MD vs. Sh + MD, *p* < 0.05). An increase in MD-induced damage was also recorded by comparing sham-exposed and MD-treated samples in presence and in absence of autophagy inhibitors (42% increase in Sham + MD with inhibitors vs. Sham + MD W/O inhibitors, *p* < 0.05).

### 2.2. SH-SY5Y CRISPRed Cells for ATG7 and ATG5 Fail to Stimulate Autophagic Flux

**Figure 2:**
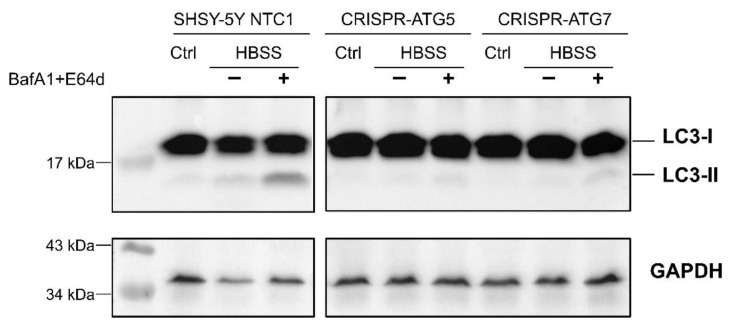
Autophagic flux in parental SH-SY5Y cells compared to their derived autophagy-deficient clones. Cells were either left untreated (Ctrl) or transferred to HBSS for 3 h to induce autophagy; to measure autophagic flux, autophagy inhibitors bafilomycin A1 and E64d were added to HBSS or not 1 h before harvesting the cells; total proteins were extracted, and LC3 conversion to its lipid-conjugated form LC3-II was monitored by Western blot. GAPDH was used as a loading control.

In Figure 2, we verified that SH-SY5Y cells are competent for autophagy: we exposed the parental cells (left panel) to nutrient starvation for 3 h (HBSS incubation) and measured the autophagic flux. In the presence of autophagy inhibitors, we found that LC3 accumulated under its PE-conjugated form (LC3-II), which testifies to the protein being anchored to autophagosomes and that autophagy is thus fully functional. Autophagy-deficient clones derived from SH-SY5Y were generated, where *ATG5* or *ATG7* genes were edited with the CRISPR method and validated by Western blot and gene sequencing provided as Supplementary Material (Figure S1). These clones were exposed to the same treatment as their parental counterpart (Figure 2, right panel). We found that LC3-II failed to accumulate, confirming that CRISPR-driven edition of *ATG5* or *ATG7* genes indeed produced autophagy incompetent cells.

### 2.3. RF-Induced AR Is Negated in ATG7 and ATG5 CRISPR Cell Lines

Cell viability was assayed in all the experimental cultures set up with SH-SY5Y CRISPR cell lines defective for *ATG7* or *ATG5* genes or control cells (NTC1). Unchallenged cells exhibited a viability higher than 80% (data not shown).

**Figure 3:**
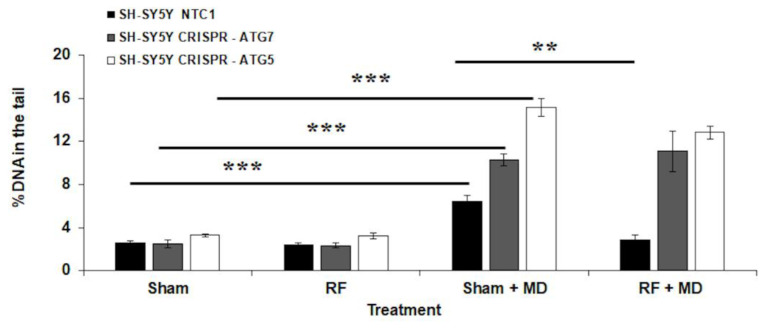
% DNA in the tail in SH-SY5Y CRISPR (NTC1, ATG7, and ATG5) cells exposed to RF for 20 h at 0.3 W/kg SAR. For each cell line, the following samples were analyzed: sham-exposed (Sham), RF-exposed at 0.3 W/kg (RF), sham-exposed and treated with MD (Sham + MD), RF-exposed and treated with MD (RF + MD). Each data point represents the mean ± SE of four (NTC1 and CRISPR-ATG7) or three (CRISPR-ATG5) independent experiments. ** *p* < 0.01; *** *p* < 0.001 (two-tailed unpaired Student’s *t*-test).

Figure 3 presents the % of DNA in the tail (mean ± SE) in the different experimental cultures as measured in the deficient SH-SY5Y cells for *ATG7* and *ATG5* genes. In all cases, RF exposure did not induce DNA damage since values of % DNA in the tail for sham and RF cultures were similar to those observed in wild-type cells. In all cases, MD treatment induced a statistically significant increase in %DNA in the tail (Sham + MD vs. Sham, *p* < 0.001). In addition, the damage induced by MD treatments (Sham + MD) was higher in CRISPR-ATG7 and CRISPR-ATG5 cells than in NTC1 control cells (62% increase, *p* < 0.001 and 137% increase, *p* < 0.001, for CRISPR-ATG7 and CRISPR-ATG5, respectively).

In response to the adaptation protocol, NTC1 cells showed AR. In particular, in four independent experiments, a 55% decrease in % DNA in the tail was recorded in RF + MD cultures with respect to Sham + MD ones (*p* < 0.01), which was of the same order of magnitude as the one recorded in SH-SY5Y wild type (49% decrease). In contrast, AR was not induced in cultures set up with both CRISPR-ATG7 (four independent experiments) and CRISPR-ATG5 (three independent experiments) cells (RF + MD vs. Sham + MD, *p* > 0.05 in both cases).

## 3. Discussion

We have previously demonstrated the pre-exposure of SH-SY5Y neuroblastoma cells for 20 h to 1950 MHz, UMTS signal, at a SAR level of 0.3 W/kg was able to induce AR by reducing the MD-induced DNA damage, most probably by enhancing antioxidant scavenging efficiency and restoring DNA repair capability [11].

Autophagy is a catabolic pathway activated in response to different cellular insults, which can range from a lack of nutrients or growth factors to the accumulation of ROS and DNA damage. It is regulated by a wide range of proteins, maintains metabolic homeostasis, and ensures the adaptation of the cells to changing environmental conditions [37,38]. Moreover, autophagy seems to be involved in DNA damage response; although, the precise role is still not well known [39].

We therefore hypothesized that the protective effect of the AR we observed in SH-SY5Y cells challenged by MD could result from a priming of autophagy by RF pre-exposure. 

In the literature, few studies have investigated the autophagy induced by RF exposure with comparable exposure parameters in different mammalian cell cultures, and the majority of them report on an enhanced autophagic flux.

An increased expression of autophagic markers, LC3-II and Beclin 1, was detected in primary rat spiral ganglion neurons pre-treated with lipopolysaccharide and exposed for 24 h (5 min on/10 min off cycles) to 1800 MHz, GSM talk mode, at 4 W/kg SAR level [32].

A couple of papers from two independent research groups pointed out that the enhancement of the autophagy flux induced in mouse spermatocyte-derived cells by the RF exposure to 1800 MHz, GSM talk mode, may have an important protective role against cellular damage, and thus provide a reasonable explanation for the RF-induced AR. In the first paper, cells exposed for 24 h at SAR levels of 1, 2, and 4 W/kg exhibited a dose-dependent increase in the expression of LC3-II, which became significant at 4 W/kg SAR. These results were also confirmed by means of GFP-LC3 transient transfection assay and transmission electron microscopy analysis [29]. In the second paper, exposure of cells for 24 h at 4 W/kg SAR also induced autophagy when the expression of LC3II/LC3I, the autophagic vacuoles, and the GFP-LC3 dots were analyzed. Moreover, the authors observed that RF exposure induced DNA damage, which significantly increased following autophagy inhibition [28]. 

Zielinski and co-workers investigated the effect of intermittent (2 min on/2 min off cycles) exposure to 935 MHz, GSM talk, for 2 and 24 h at 4 W/kg SAR on autophagy in SH-SY5Y and in microglial (N9) cells. Protein levels of the autophagy marker ATG5 significantly increased after 24 h RF exposures in neuroblastoma cells but not in N9 cells, evidencing the dependency of the effect on cell type and exposure duration [31]. 

Apart from articles investigating the relationship between RF and macro-autophagy, Terro and co-workers evaluated the link with chaperone-mediated autophagy (CMA), a process genetically distinct from macro-autophagy. While macro-autophagy is reported to be unselective, CMA is instead selective for proteins harboring a KFERQ motif. HSC70 guides these proteins for direct lysosomal import through LAMP2A. Terro et al. investigated CMA in primary cerebral cortical cell cultures exposed for 24 h to 900 MHz (GSM signal) at 0.25 W/kg SAR. They studied the expression of LAMP-2A, HSC70, HSP40, and HSP90, molecular actors of CMA, and the level of α-synuclein, a CMA substrate. They found an increased HSC70, a decreased HSP90, and a decrease in α-synuclein, while the levels of HSP40 and HSP70 remained unchanged. Since comparable effects were detected in cells incubated at 37.5 °C, a condition that mimics the GSM-generated temperature rise they measured under their experimental conditions, the authors concluded that the observed effects were likely due to temperature rise in exposed cell cultures [30].

Building up from these considerations, we employed the experimental setup that we previously showed to drive adaptation in SH-SY5Y cells, to test the involvement of autophagy in the RF-induced AR against MD treatment. We found the negation of AR when autophagy was blunted, both by chemical inhibitors and as a result of the CRISPR-driven genetic edition of *ATG* genes. These observations of the negation of RF-induced AR following both chemical and genetic inhibition of autophagy, which strengthen each other, pointed out a possible role of this catabolic cellular pathway. It should be highlighted that the exposure system we employed here is strictly controlled in terms of dosimetry and temperature; thus, we can exclude thermal effects.

Moreover, it has been recognized that autophagy promotes DNA repair and participates in DNA damage response and cell fate decision; although, its role and the involved pathways are still under investigation [40,41]. Consistent with this observation, we evidenced here an increase in the MD-induced DNA damage following inhibition of autophagy, both in cells treated with inhibitors and in ATG7 and ATG5 knocked cells.

Then, the negation of AR as a consequence of autophagy inhibition can further support the involvement of DNA repair mechanisms in the phenomenon, which has been demonstrated previously in other cell types via direct and indirect measurements of poly (ADP-ribose) polymerase enzyme (PARP-1) mRNA expression, and its protein level [14,21,22]. PARP involvement is also referred to as one of the possible mechanisms underneath the ionizing radiation-induced AR [42,43].

If direct evidence of the role of autophagy is provided in further investigations, another analogy with the low-dose ionizing radiation-induced AR will be highlighted. As a matter of fact, the involvement of autophagy could add to the evidence already provided on the role of DNA damage repair and antioxidant response highlighted for RF and ionizing radiation-induced AR [34]. 

As a whole, the results here presented to our knowledge demonstrate that autophagy is clearly required for the onset of an RF-induced adaptive response. This is the solid platform we needed to build from to explore, in a future study, the signaling pathways involved. Moreover, our findings provide another piece of information towards the understanding of cellular mechanisms underneath the RF-induced AR.

## 4. Materials and Methods

### 4.1. Reagents 

Dulbecco’s modified Eagle’s medium (DMEM) 4.5 g/L Glucose and fetal bovine serum (FBS) were from Dominique Dutscher (Brumath, France), Glutamax was from Life Technologies (Milano, Italy), trypsin-EDTA and penicillin/streptomycin were from Biowhittaker (Verviers, Belgium). Triton X-100, N-lauryl sarcosine, MD, Bafilomycin A1, and E64d-protease inhibitors were from SIGMA (St. Louis, MO, USA). Dimethyl sulfoxide (DMSO), NaOH, and Na_2_EDTA were from Baker (Deventer, The Netherlands). Tris and NaCl were from Carlo Erba (Milan, Italy). Normal-melting-point agarose, low-melting-point agarose, and ethidium bromide were from Bio-Rad Laboratories (GmbH, Munich, Germany). Nitrocellulose membranes for Western blotting were AmershamProtran™ 0.2 µm NC, Chicago, IL, USA. Antibodies used are rabbit anti-LC3 (#L7543, Sigma Aldrich, St. Louis, MO, USA), anti-ATG5 (A0731, Sigma Aldrich), Anti-ATG7 (clone D12B11, Cell signaling Technology, Danvers, MA, USA), and horseradish peroxidase-conjugated secondary antibodies from Jackson Immunoresearch. NP-40 was from Fisher Scientific (Illkirch, France). Western blots were revealed with Clarity Western ECL (Bio-Rad laboratories, Marnes-la-Coquette, France). Chemiluminescence was detected with a G:Box imaging system (Syngene, Cambridge, UK).

### 4.2. Exposure System Set Up and Dosimetry

The exposure setup consisted of an RF generator (E4432B ESG-D, Agilent, Santa Clara, CA, USA), which provides the 1950 MHz, UMTS signal, a microwave amplifier (AM38A-0925-40-43), a 6 dB power splitter (HP11667A, Hewlett-Packard, Palo Alto, CA, USA), and two bidirectional power sensors (NRT-Z43, Rohde & Schwarz, Munich, Germany). The output signals of the power splitters were sent, through the power sensors, to two identical WR430 (350 mm long, SAIREM), short-circuited waveguides, connected to the feeding side by means of a coaxial-to-waveguide adapter (Maury Microwave R213A2, VSWR: 1.05). The signal generator and the power sensors were remotely controlled by a PC in a feedback loop, employed to continuously monitor the incident and reflected power levels, and to adjust them to keep the required SAR constant.

The two waveguides were placed inside a cell culture incubator (to guarantee a 37 °C, 95% air, and 5% CO_2_ atmosphere), together with a third one, used for sham exposures.

The waveguide applicators were optimized through numerical and experimental dosimetry to obtain high efficiency (>70%) and uniformity of SAR distribution (coefficient of variation <30%) in the biological samples at 1950 MHz [44].

By exploiting the symmetry of the unperturbed fundamental mode transverse electric TE_10_, as well as that of the sample container, up to 4 samples can be exposed simultaneously to two different SAR values. By using a four-layer Plexiglas stand, the relative vertical distances between the samples were set in such a way to obtain a SAR ratio of 1:4 between the central and the distal couple of samples. In our experiments, the average SAR was 1.25 W/kg in the central samples (dummy cultures) and 0.3 W/kg in the distal samples (cell cultures). To rule out heating-induced effects, temperature measurements were carried out at regular 5 s intervals for 20 h (accuracy of ±0.3 °C) in separate experiments, using a fiber-optic thermometer (FisoUMI4, FISO Technologies, Quebec City, QC, Canada) with a fiber-optic temperature probe (FISO Technologies, FOT-M/2 m) inserted horizontally into the culture medium. In five independent measurements, the temperature never exceeded the instrument sensitivity (±0.3 °C).

### 4.3. Cell Models and Culturing

#### 4.3.1. SH-SY5Y Neuroblastoma Cell Line—Wild Type

Human SH-SY5Y neuroblastoma cell line was obtained from the American Type Culture Collection (ATCC, Cat. No. CRL2266, Rockville, MD, USA). A master bank of cells was established and the same batch of reagents was used to ensure consistency and reproducibility among the experiments. Cells were cultured in DMEM 4.5 g/L glucose, supplemented with 10% heat-inactivated FBS, 1% Glutamax, 100 U/mL penicillin, and 100 mg/mL streptomycin and maintained in a 5% CO_2_ humidified atmosphere at 37 °C (commercial incubator, model 311, Forma Scientific, Freehold, NJ, USA). Cultures were kept exponentially growing by splitting them once a week by trypsin treatment, and were tested regularly for mycoplasma contamination. Cells from passages 3 to 10 were used for the experiments, and cells harvested from the same parent flask were used for each experimental run.

#### 4.3.2. SH-SY5Y—CRISPR Cell Lines

The Clustered Regularly Interspaced Short Palindromic Repeats (CRISPR) system was used as a genome editing method to delete *ATG7* or *ATG5* autophagy gene in SH-SY5Y cells. A constitutively active CRISPR/CAS9 system was chosen, and the lentiCRISPR vectors containing either a gRNA sequence targeting *ATG7* and *ATG5* or a non-targeting control sequence (NTC1), were those described by [45]. The host packaging cell line HEK 293T was used for the production of lentiviruses encoding CAS9 and the respective gRNAs. SH-SY5Y cells were then infected and submitted to antibiotic selection for two weeks. A clonal dilution was then performed by flow cytometry, and after amplification, several clones were verified by Western blot analysis for *ATG7* and *ATG5* deletion, and further confirmed by nucleotide sequencing of *ATG7* and *ATG5* genes.

SH-SY5Y CRISPR cell cultures were maintained and handled under the same conditions reported for the wild-type.

### 4.4. Experimental Procedures

The induction of AR in wild-type SH-SY5Ycells was tested in presence and in absence of the lysosomal ATPase inhibitor bafilomycin A1 (0.1 µM), and the cystein protease inhibitor E64d (30 µM), which are both widely used autophagy inhibitors, and in CRISPR cells defective for *ATG7* and *ATG5* and in their control cells (NTC1). 

Forty-eight hours before the experiments, 3 mL cultures were set up by seeding 8 × 10^5^ cells in 35 mm coded Petri dishes (Corning, catalogue no. 430165, New York, NY, USA), and grown for a total of 72 h. RF at 1950 MHz, UMTS signal, was given continuously for 20 h (from 48 to 68 h after cell seeding) at 0.3 W/kg SAR, as in Table 1. MD was dissolved in DMSO immediately before treatments and added 3 h after the end of RF/sham exposure at final concentration of 10 µM. 

Four independent experiments were carried out in wild-type cells, and for each experimental run, eight randomly assigned cultures were set up and handled in parallel to test, namely, sham-exposed (Sham), RF-exposed (RF), sham-exposed, and MD treated (Sham + MD), and RF-exposed and MD treated (RF + MD) conditions, in presence and in absence of autophagy inhibitors, added at 71 h. [46].

When CRISPR ATG cells were used, 4 independent experiments on NTC1 and ATG7 cells, and 3 independent experiments on ATG5 cells, were carried out. In this case, four cultures were set up to test Sham, RF, Sham + MD, and RF + MD.

All the analyses were performed in blind, i.e., the researchers involved in sample processing were not aware of the exposures/treatments, and data were decoded after completion of analyses.

### 4.5. Assay Procedures

#### 4.5.1. Western Blot

Total proteins were extracted in RIPA buffer (100 mM tris–HCl pH 7.4, 0.5% NP-40, 0.5% sodium-deoxycholate, 0.1% SDS supplemented with proteases inhibitor Mini^®^) for 20 min. The solubilized proteins were then recovered in the supernatant after a 20 min centrifugation at 12,000× *g*. and their concentration assayed with BCA protein assay kit. Total proteins were separated by electrophoresis on 12.5% SDS-polyacrylamide gels and transferred onto nitrocellulose-blotting membranes. Blocking solution was 3% nonfat milk in PBS-tween. Antibodies used were rabbit anti-LC3 and horseradish peroxidase-conjugated secondary antibodies. Western blots were revealed with Clarity Western ECL. Chemiluminescence was detected with a G:Box imaging system.

#### 4.5.2. Alkaline Comet Assay

The induction of DNA strand breaks and alkali labile sites was analyzed by the alkaline comet assay according to Singh et al. [47], with further modifications to obtain a consistent DNA migration in the control cells and a subsequent higher sensitivity [48]. 

After trypsinization, about 1 × 10^5^ cells were suspended in 100 μL low-melting point agarose (LMA, 0.5% *w*/*v*), and sandwiched between a lower layer of normal melting point agarose (NMA, 1% *w*/*v*) stratified on a microscopy slide, and an upper layer of LMA (0.5% *w*/*v*). The slides were incubated in freshly prepared cold lysis solution (2.5 M NaCl, 100 mM Na2EDTA, 10 mM Tris, 25 mM NaOH, pH 10, 1% N-lauryl sarcosine, 1% Triton X-100 and 10% DMSO) for 60 min at 4 °C and, after 40 min unwinding at 4 °C, slides were electrophoresed at 30 V, 340 mA for 40 min at 4 °C in electrophoresis buffer (300 mM NaOH, 1 mM Na2EDTA, pH 13). Finally, slides were neutralized by three changes of neutralizing buffer (0.4 M Tris-HCl pH 7.5) and left in distilled water for 5 min. Slides were air-dried and stained just before analysis with 12 μg/mL ethidium bromide. DNA damage was scored on two replicate slides for each condition, and images of 500 randomly selected nuclei (250 from each slide) were analyzed using a computerized image analysis system (Delta Sistemi, Rome, Italy) fitted with a Leica DM BL fluorescence microscope (Leica Microsystems, Mannheim, Germany) at 200× magnification. The system acquires images and evaluates the percentage of DNA migrated in the tail as a measurement of DNA integrity [49].

### 4.6. Statistical Analysis

All data are represented as the mean ± SE. Unpaired Student’s t test was used to analyze Comet assay data. A threshold of *p* < 0.05 was set as statistically significant.

## 5. Conclusions

This study aimed to investigate whether autophagy has a role in RF-induced AR in SH-SY5Y cells pre-exposed to 1950 MHz, UMTS signal, 0.3 W/kg SAR, and then treated with MD. By applying the Comet assay, the reduction in the MD-induced DNA damage was evaluated in SH-SY5Y wild-type cells, in presence and in absence of autophagy inhibitors, as well as in defective ATG cell lines. The results obtained indicate that AR was negated both when autophagy was inhibited by bafilomycin A1 and E64d, and when CRISPR cell lines defective for *ATG7* or *ATG5* genes were used. The evidence here presented on the negation of RF-induced AR, after both chemical and genetic inhibition of autophagy, highlights a possible role of this catabolic cellular pathway in the cell model here used, and lays the ground to provide direct evidence of the role of autophagy in RF-induced AR, provided that the same results are also confirmed in healthy cell cultures.

## Figures and Tables

**Table 1:** Exposure conditions employed in the experiments.

Frequency	Signal	SAR	Exposure Duration
1950 MHz	UMTS	0.3 W/kg	20 h

## Data Availability

Not applicable.

## Supplementary Materials

The following supporting information can be downloaded at: https://www.mdpi.com/article/10.3390/ijms23158414/s1.
