# Supplementary material for: Inhibition of Autophagy Negates Radiofrequency-Induced Adaptive Response in SH-SY5Y Neuroblastoma Cells
Source: Int J Mol Sci. 2022 Jul 29;23(15):8414. doi: 10.3390/ijms23158414 (PMC9369083; doi:10.3390/ijms23158414)
Supplement: Supplementary file 1 [file ijms-23-08414-s001.zip › ijms-1774327-supplementary.pdf]

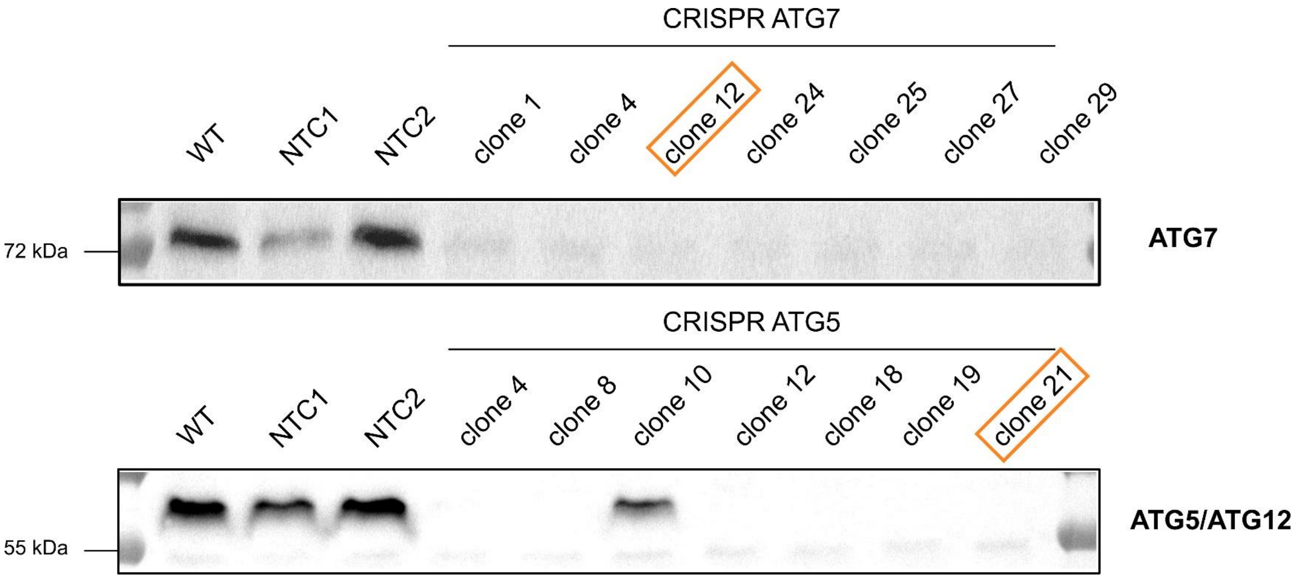


**Figure S1.** Selection and validation of autophagy-deficient SH-SY5Y clones: ATG5 and ATG7 genes were targeted for CRISPR-mediated gene edition in SH-SY5Y cells as indicated in the material and method section. After puromycin selection, individual cells were plated through cytometry sorting, and the resulting clones were then amplified and submitted to western blot and gene sequencing analyses (not shown) to validate that the targeted genes were no longer expressed. Clone 12 was selected for SH-SY5Y CRISPR ATG7 cells, and clone 21 was selected for SH-SY5Y CRISP ATG5 cells.
